# Navigating Through the Rare and Accepting the New Shift: Laparoscopic Gastro-Jejunostomy for Gastric Outlet Obstruction Secondary to Eosinophilic Gastritis in a Pediatric Patient

**DOI:** 10.7759/cureus.65540

**Published:** 2024-07-27

**Authors:** Ajinkya Akre, Santosh Thorat, Akshay C Mhase, Preet V Shah

**Affiliations:** 1 General Surgery, Pimpri Chinchwad Municipal Corporation's Postgraduate Institute Yashwantrao Chavan Memorial Hospital, Pune, IND; 2 Surgical Gastroenterology, Pimpri Chinchwad Municipal Corporation's Postgraduate Institute Yashwantrao Chavan Memorial Hospital, Pune, IND; 3 Surgery, Pimpri Chinchwad Municipal Corporation's Postgraduate Institute Yashwantrao Chavan Memorial Hospital, Pune, IND

**Keywords:** hydatid cyst, histopathologic diagnosis, gastroenterology and endoscopy, unknown etiology, infectious and tropical diseases, laparoscopic treatment, pediatric gastroenterology, pyloric stenosis, eosinophilic gastrointestinal disease, minimal access surgery

## Abstract

Eosinophilic gastritis, a rare variant of gastritis, presents with inflammation of the stomach lining due to eosinophil infiltration. This case report describes a complex presentation of eosinophilic gastritis in a 12-year-old boy, highlighting the challenges encountered in management. A 12-year-old male presented with symptoms consistent with gastritis, including abdominal pain, nausea, and vomiting. Despite extensive medical workup to identify potential etiologies (parasitic infections, autoimmune conditions), the diagnosis of eosinophilic gastritis was established. Unfortunately, the patient exhibited persistent symptoms despite aggressive medical management. The case was further complicated by pyloric stenosis, a narrowing of the stomach outlet. Laparoscopic intervention, a minimally invasive surgical approach, was initially attempted but deemed challenging due to the patient's specific condition. The presence of metabolic abnormalities added further complexity. Alternative approaches, such as endoscopic dilatation, were considered but ultimately deemed unsuitable due to the severity of the stenosis and the desire for a minimally invasive solution compared to laparotomy. This case exemplifies the challenges associated with managing rare gastrointestinal conditions like eosinophilic gastritis, particularly in pediatric patients. The report emphasizes the importance of a multidisciplinary approach, involving collaboration between gastroenterologists, surgeons, and potentially other specialists depending on the specific complications, to achieve optimal outcomes. This case highlights the complexities in managing this patient, especially when accompanied by complications like pyloric stenosis. It underscores the crucial role of a multidisciplinary team in navigating challenging presentations and exploring minimally invasive surgical options when feasible.

## Introduction

Eosinophilic gastrointestinal (GI) disorders encompass conditions marked by abnormal eosinophil accumulation in the GI tract, including eosinophilic esophagitis, eosinophilic gastritis, eosinophilic enteritis, and eosinophilic colitis. The prominent histological features of eosinophilic gastritis include eosinophil accumulation and basal layer expansion in the epithelium, along with fibrosis of the lamina propria [1].

Limited research suggests a potential link between various stimuli and the initiation of inflammatory processes. These stimuli include food allergies, pre-existing inflammatory conditions, infectious agents, malignancies, and specific medications such as gold therapy, azathioprine, enalapril, carbamazepine, and antitumor necrosis factor agents [2,3].

Eosinophilic gastritis, exhibiting a prevalence of 6.3 per 100,000 individuals [4], is orchestrated by a complex and intricate interplay of cytokines and chemokines, including interleukin-3 (IL-3), IL-5, and granulocyte-macrophage colony-stimulating factor. These mediators are instrumental in the proliferation and activation of eosinophils [5].

The Klein classification system serves as a means to categorize disease types based on the specific layer involved. The system delineates three primary categories: mucosal, muscular, and serosal [6].

In this report, we present a rare instance of critical pyloric stenosis in a 12-year-old boy. This condition arose secondary to eosinophilic gastritis. The patient's presentation included nausea, vomiting, early satiety, and abdominal pain. To confirm the diagnosis of gastric outlet obstruction and eosinophilic gastritis, abdominal computed tomography and esophagogastroduodenoscopy with biopsy were employed. Initial treatment with high-dose systemic corticosteroids and the treatment for eradication of helicobacter pylori proved ineffective, necessitating a laparoscopic gastrojejunostomy. This surgical intervention created a new passage between the stomach and the small intestine, bypassing the obstructed pylorus.

While the specific cause (etiology) of eosinophilic gastritis in this patient remains undefined, this prompt diagnosis, followed by a successful laparoscopic gastrojejunostomy, not only provided relief for the patient but also offered valuable insights into the presentation and management of eosinophilic gastritis in pediatric patients. Analyzing such cases can improve our understanding of this complex disease and its potential complications in a younger population.

## Case presentation

We present a case of a 12-year-old male patient who presented to the emergency department with chief complaints of epigastric abdominal pain. The pain specifically localized to the upper central region of the abdomen and occurred predominantly following meals. Additionally, the patient reported experiencing multiple episodes of vomiting, 4-5 times a day. The vomitus contained food particles and was not bilious. The patient's medical history revealed prior hospital admissions. On general examination, the patient exhibited signs of dehydration, including a dry tongue, sunken eyes, and poor skin turgor.

The patient was admitted to the intensive care unit and on admission arterial blood gas analysis showed an elevated pH of 7.61, HCO3- of 58, potassium(K+) of 3.1, chloride (Cl-) of 72, a base deficit of 58.2, sO_2_ of 99.3 percent, and lactate of 1.7 mmol/L suggestive of mild metabolic hypochloremic alkalosis. Laboratory evaluation revealed the following results: a white blood cell count of 15,200 cells/mm³ (reference range: 3,300 to 9,400 cells/mm³) with an eosinophil count of 9200 cells/mm³ (reference range: 0 to 564 cells/mm³), and serum albumin level of 3.6 g/dL (reference range: 4.3 to 5.4 g/dL). Serum IgE levels were within normal limits. Stool studies for ova and parasites, as well as stool cultures, were negative.

**Figure 1:**
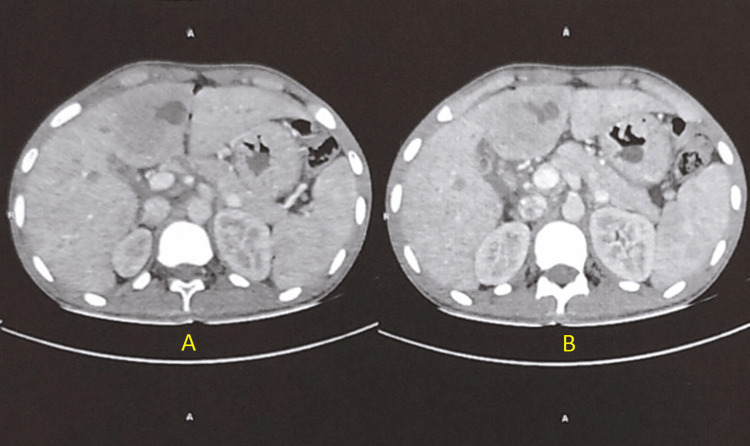
Sagittal section of CECT abdomen and pelvis showing a dilated stomach with fluid residue and multiple liver lesions Panel A shows well-defined oval cystic lesions in segment IVB and segment III of the liver. Panel B shows a dilated stomach with fluid residue. CECT: Contrast-enhanced computed tomography

Other lab investigations were unremarkable. The contrast-enhanced computed tomography scan suggested well-defined oval shaped solid cystic lesions which were suspected hydatid cysts of size 2.1x2x2.3 cm in segment IVB, 0.7x0.7cm lesion in segment III of the left lobe and 2x1.8x2cm lesion in segment V and VIII of the right lobe of the liver as shown in Figure 1. 

**Figure 2:**
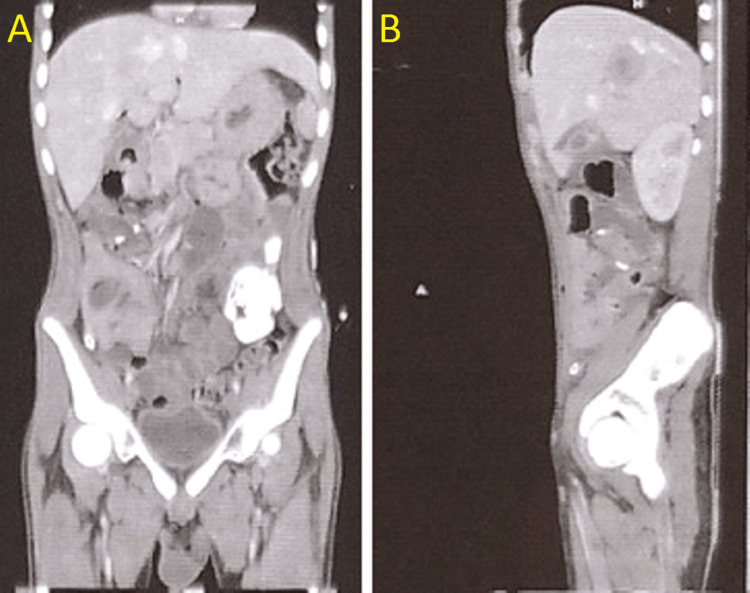
Coronal section of the contrast-enhanced computed tomography scan of abdomen pelvis showing multiple liver lesions Panel A shows a lesion in segment V of the liver. Panel B shows a lesion in the liver with central cystic and peripheral solid components.

The lesion shows peripheral solid areas with a central cystic component shown in Figure 2. The patient had been on ongoing anti-helminthic therapy for the past six months for the same. A comparison of earlier computed tomography scans showed regression of lesions indicating response to albendazole.

On admission, he was managed conservatively for one week with correction of metabolic abnormalities and was given a cold saline wash through a nasogastric tube. Vomiting reduced to 1-2 episodes per day; however, there was no complete resolution of symptoms. Following this, the patient was subjected to oesophago-gastro duodenoscopy.

**Figure 3:**
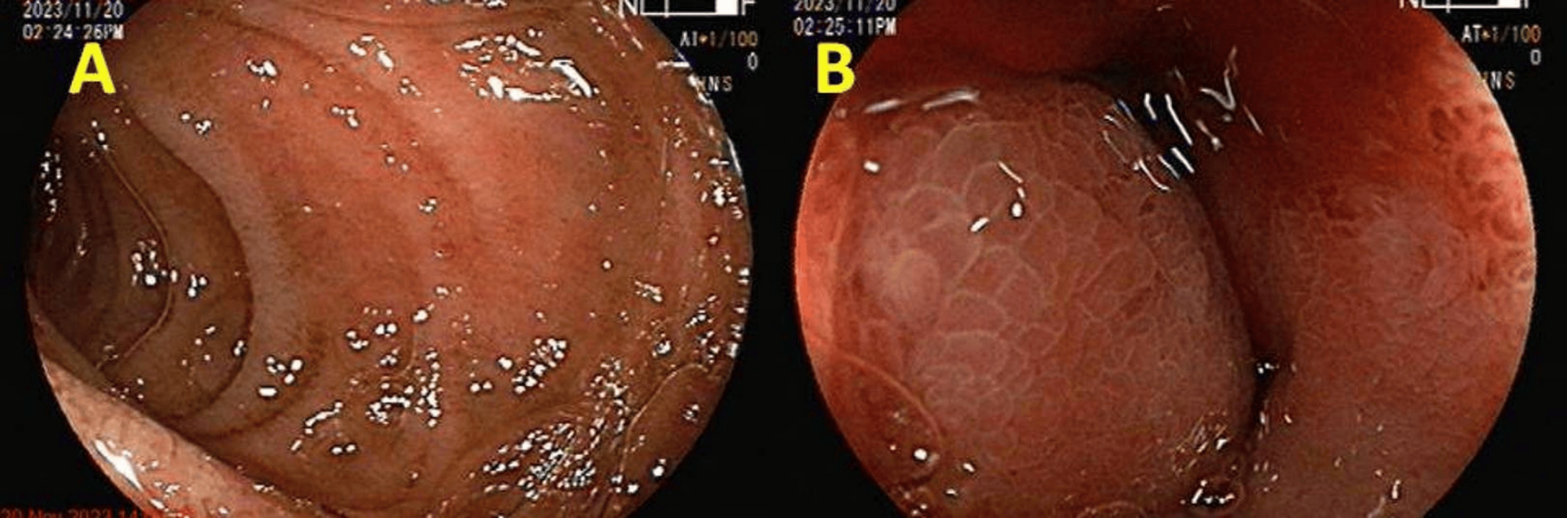
Endoscopy image showing a grossly dilated stomach and nodular, erythematous, and friable mucosa Panel A shows a grossly dilated stomach. Panel B shows friable, erythematous, and nodular mucosa.

Findings revealed changes of severe GERD at the oesophago-gastric junction and a grossly dilated stomach with a lot of fluid residue, inflammation, and erythema of stomach mucosa shown in Figure 3.

**Figure 4:**
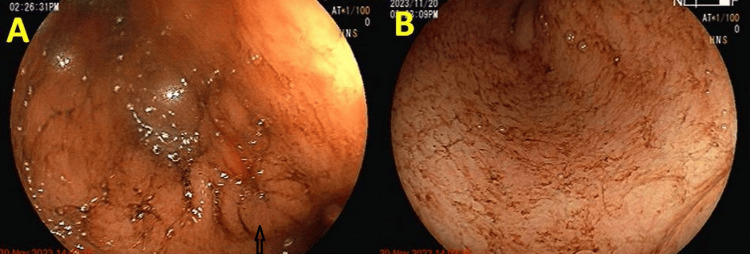
Endoscopy image showing prepyloric bulge of pyloric mucosa in antrum indicative of pyloric stenosis and fluid residue in the stomach Obliterated pylorus marked by the black arrow Panel A shows narrowed and deformed pylorus causing gastric outlet obstruction. Panel B shows residual fluid in the body of stomach as a result of gastric outlet obstruction.

The pylorus was narrowed and deformed and gastric outlet obstruction was evident, due to which passing scope was difficult which ruled out the option for stenting in pyloric stenosis. The endoscopic image is shown in Figure 4.

**Figure 5:**
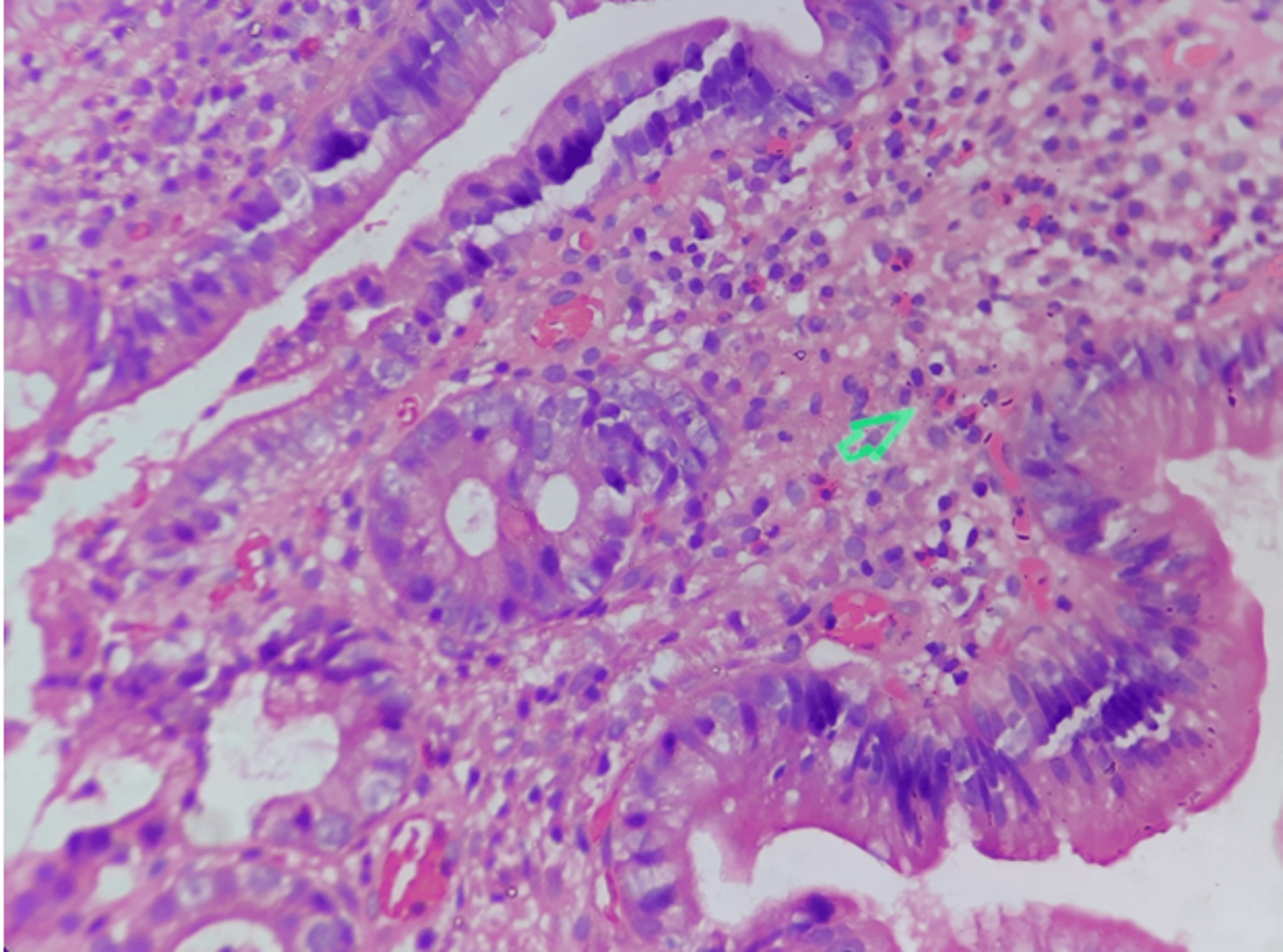
Histopathology image showing invasion of eosinophils into the muscular layer of the stomach The green arrow marks the eosinophils.

Biopsy confirmed eosinophilic infiltration in the antrum and pylorus and thus eosinophilic gastritis was confirmed with invasion of eosinophils into the muscularis layer of the stomach. The microscopic histopathology image is shown in Figure 5.

Prednisolone was started with an initial dose of 0.5mg/kg/day and increased up to 0.8mg/kg/day.

As the most accepted treatment for H. pylori eradication in pediatric age group is first-line triple therapy, Omeprazole 1 mg/kg, before breakfast, amoxicillin 50 mg/kg BD after meals, and clarithromycin 15 mg/kg BD were given for seven days.

However, there was no symptomatic improvement in clinical as well as radiological findings. In light of enduring symptoms, the decision was made to proceed with surgical intervention.

A laparoscopic Roux-en-Y gastrojejunostomy (LRYGJ) was performed to address the patient's condition. To facilitate the procedure, a quadrilateral laparoscopic access was established using four trocars. Two 5 mm trocars were inserted in the left upper quadrant, one in the left iliac fossa and the other in the left paraumbilical region. A 10 mm camera port was placed in the subumbilical region and a 12 mm working port was placed in the right upper quadrant.

**Figure 6:**
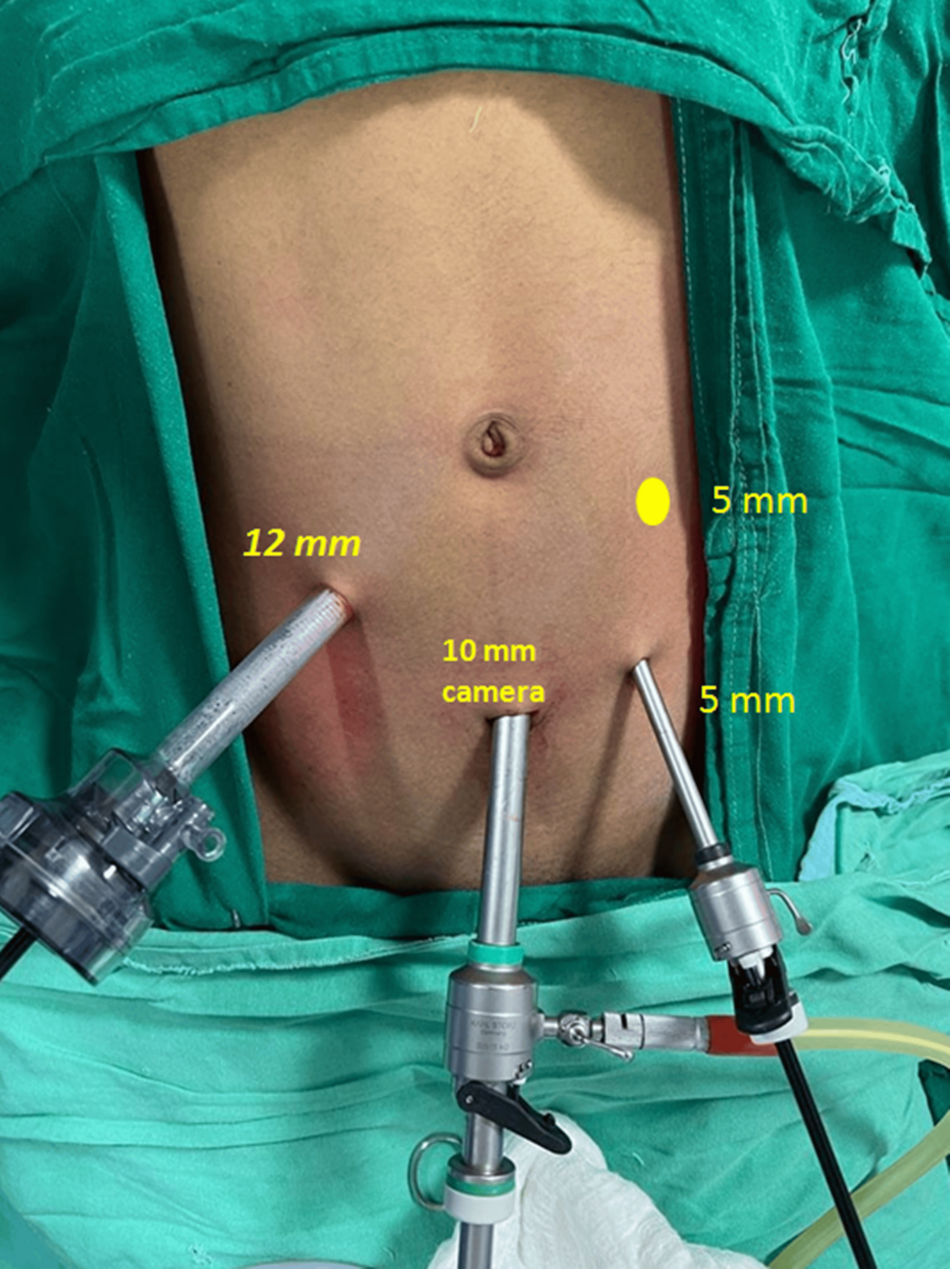
Abdomen showing laparoscopic port placements

Figure 6 shows the positioning of the laparoscopic ports.

**Figure 7:**
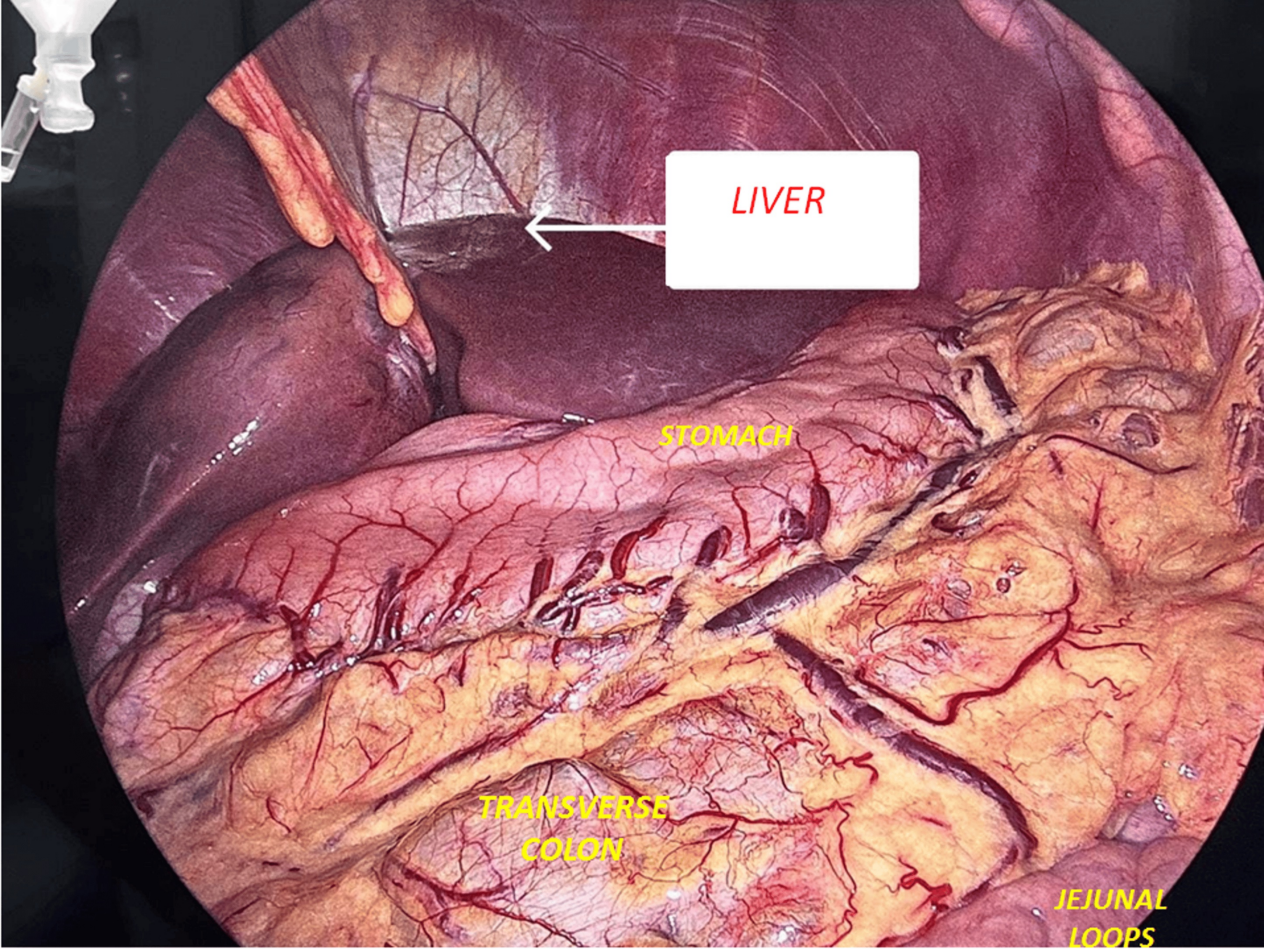
Diagnostic laparoscopy image showing normal liver parenchyma, stomach, transverse colon, and jejunal loops

 Diagnostic laparoscopy revealed no gross abnormalities of the liver parenchyma as shown in Figure 7.

**Figure 8:**
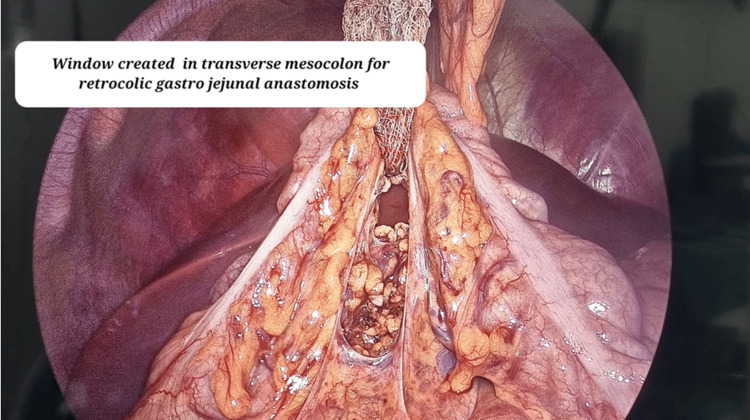
Laparoscopic image showing window created in the transverse mesocolon for gastrojejunal anastomosis

The greater omentum was dissected from the greater curvature of the stomach using bipolar electrocautery. A window was created in the transverse mesocolon to facilitate a retrocolic gastrojejunal anastomosis as shown in Figure 8.

The jejunum was transected approximately 40 cm distal to the duodenojejunal flexure. Enterotomies were created on the free distal jejunal loop and the antrum of the stomach.

A side-to-side gastrojejunal anastomosis was performed using an endostapler. The enterotomy sites were created for the insertion of the two limbs of the endostapler for anastomosis.

**Figure 9:**
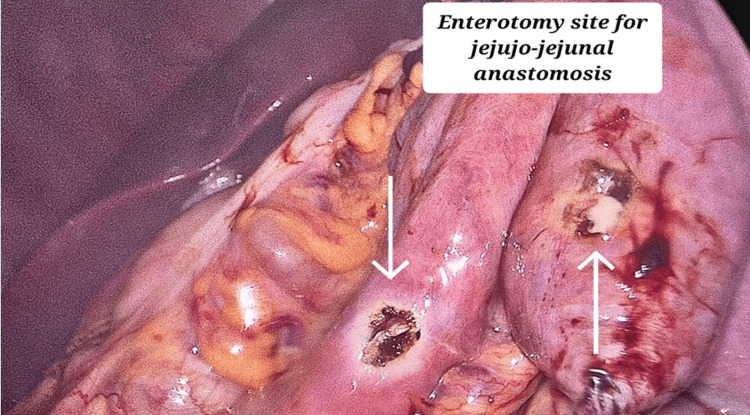
Laparoscopic image showing entorotomy sites created on the proximal and the distal jejunal loop for jejuno-jejunal anastomosis

For a side-to-side jejunojejunal anastomosis, enterotomy sites were created in the proximal jejunal limb (containing biliopancreatic secretions) and the distal jejunal limb as shown in Figure 9 and the anastomosis was performed using an endostapler. 

All dissections, transections, and anastomoses were performed using an endoscopic stapler as shown in Video 1. The free ends after the gastro-jejunal and the jejuno-jejunal anastomosis were sutured with absorbable polydioxanane sutures. Patency of gastro-jejunal anastomosis and watertight repair were confirmed with leak test by passing air from ryles tube with occlusion of distal bowel with help of bowel holding forceps as shown in Video 1. Abdominal drain was kept in situ.

**Video 1:** Video showing the jejunal transection, gastro-jejunal anastomosis and the leak test to confirm patency of GJ anastomosis

Post-operatively, the patient was kept in the intensive care unit with strict monitoring of vitals, temperature, intake and output. IV fluids for correction were DNS 80cc/hr + 10mEq KCl per day with NS as 200ml bolus as and when required to maintain urine output at 800-1100ml per day.

At the third post-operative day (POD), the patient developed fever of 102ºF due to urinary tract infection confirmed with urine routine microscopy. The urinary catheter was changed and conservative treatment with antibiotics along with urinary alkalizer helped to alleviate the fever.

The patient tolerated a clear liquid diet starting on POD 4, coinciding with an improvement in postoperative nausea and vomiting.

On POD 5, the nasogastric tube and abdominal drain were removed due to the absence of gastrointestinal secretions and passage of stool.

The patient was transferred to the ward setting following confirmation of bowel function. Discharge occurred on POD 10 after suture removal.

## Discussion

Eosinophilic gastritis presenting with gastric outlet obstruction is an uncommon and challenging clinical entity and is recognized as a chronic inflammatory disorder [7].

Upon recruitment to the gastrointestinal tract, activated eosinophils elicit a substantial inflammatory response by releasing an array of mediators, inclusive of cytotoxic granules. These granules contribute to structural damage within the infiltrated layers of the tissue [8].

In our patient, imaging studies revealed findings of small liver lesions, potentially consistent with transitional and degenerative hydatid cysts belonging to the CE3B and CE4 WHO classification of hydatid cysts [9]. These cysts can be a contributing factor to eosinophilic gastritis, as parasitic infections can trigger allergic reactions leading to eosinophil infiltration in the stomach lining [10]. Given the challenging accessibility of the cysts and observed regression, a non-surgical approach was adopted [11,12]. Albendazole emerged as the preferred pharmacological intervention due to its notable efficacy in inducing shrinkage or complete disappearance of the cystic structures, surpassing other therapeutic agents employed thus far [13,14]. Encouragingly, our patient demonstrated a favorable response with notable reduction in the size of the hydatid cysts.

Helicobacter pylori may contribute to the pathogenesis of eosinophilic gastroenteritis. The eradication of H. pylori may prove beneficial in the management of select cases of this rare syndrome [15]. Numerous clinical cases have indicated that eradicating Helicobacter pylori infection may be effective in the treatment of eosinophilic gastroenteritis. Consequently, bacterial eradication of H. pylori should be considered prior to the implementation of more invasive treatments [16]. Standardized Drug dosages of standard triple line therapy for the pediatric age group were used [17]. Clarithromycin is also recognized for its immunomodulatory effects, which include the inhibition of T-cell proliferation and the induction of eosinophil apoptosis [18].

Pyloric stenosis resulting in gastric outlet obstruction is categorized under the ‘complicated’ classification within the severity index of eosinophilic gastroenteritis as assessed by histological and endoscopic examination [19].

Due to the patient's acute presentation and failure to respond to conservative management, a surgical approach was undertaken. This is consistent with the reported surgical intervention rate of approximately 40% in patients with eosinophilic gastroenteritis who experience a refractory course of disease [20].

## Conclusions

Eosinophilic gastritis, although rare, is a serious condition within the spectrum of gastritis. It is caused by an abnormal immune response in the stomach lining, resulting in inflammation and tissue damage.

Parasitic infections or systemic immune diseases may be implicated as the underlying cause. However, even in individuals without apparent health issues, eosinophil infiltration, indicative of a physiologic immune response, may be observed. Research into potential genetic or environmental factors contributing to eosinophil infiltration in the stomach lining is crucial. While the underlying cause of this uncommon condition remains unclear, achieving a timely diagnosis is paramount. Studies comparing the efficacy of different treatment modalities and their optimal duration are needed. Further research is needed to understand the long-term consequences of Eosinophilic Gastritis and its potential impact on gastrointestinal health in pediatric patients as its complex and unpredictable nature necessitates a collaborative approach toward patient care. For patients with established fibrosis who don't respond to medical therapy, timely surgical intervention is essential to minimize gastrointestinal dysfunction.
